# Impact of Sacubitril/Valsartan on the Long-Term Incidence of Ventricular Arrhythmias in Chronic Heart Failure Patients

**DOI:** 10.3390/jcm8101582

**Published:** 2019-10-02

**Authors:** Ibrahim El-Battrawy, Christina Pilsinger, Volker Liebe, Siegfried Lang, Jürgen Kuschyk, Xiaobo Zhou, Martin Borggrefe, Susanne Röger, Ibrahim Akin

**Affiliations:** 1 First Department of Medicine, Faculty of Medicine, University Medical Centre Mannheim (UMM), University of Heidelberg, 68167 Mannheim, Germany; christina.pilsinger@gmail.com (C.P.); Volker.Liebe@umm.de (V.L.); siegfried.lang@umm.de (S.L.); Juergen.kuschyk@medma.uni-heidelberg.de (J.K.); Xiaobo.zhou@medma.uni-heidelberg.de (X.Z.); Martin.borggrefe2006@gmail.com (M.B.); Susanne.Roeger@umm.de (S.R.); Ibrahim.akin@umm.de (I.A.); 2 DZHK (German Center for Cardiovascular Research), Partner Site, Heidelberg-Mannheim, 68167 Mannheim, Germany

**Keywords:** life-threatening arrhythmia, sacubitril/valsartan, sudden cardiac death

## Abstract

Background: Sacubitril/valsartan decreased the risk of sudden cardiac death (SCD) in patients suffering from heart failure with reduced ejection fraction (HFrEF). However, long-term data are sparse. Objective: The aim of the present study was to compare the incidence of life-threatening arrhythmias consisting of ventricular tachycardia and/or ventricular fibrillation before and after initiation of sacubitril/valsartan treatment. Methods: Out of 12,000 patients with HFrEF from 2016–2018, 148 patients were newly prescribed sacubitril/valsartan, but the long-term data of only 127 patients were available and included in this study. Results: Patients with an average age of 66.8 ± 12.1 had a median left ventricular ejection fraction (LVEF) of 25% (interquartile range (IQR) 5.00–45.00) and 30% (IQR 10.00–55.00, *p* < 0.0005) before and after sacubitril/valsartan treatment, respectively. Systolic blood pressure decreased from 127.93 ± 22.01 to 118.36 ± 20.55 mmHg (*p* = 0.0035) at 6 months of follow-up. However, in 59 patients with a long-term outcome of 12 months, ventricular arrhythmias persistently increased (ventricular fibrillation from 27.6 to 29.3%, ventricular tachycardia (VT) from 12% to 13.8%, and nonsustained VT from 26.6 to 33.3%). Conclusions: Sacubitril/valsartan does not reduce the risk of ventricular tachyarrhythmias in chronic HFrEF patients over 12 months of follow-up.

## 1. Introduction

The mortality rate of patients suffering from chronic heart failure is still high despite advantages in diagnosis and treatment. Recently, promising data from the administration of sacubitril and valsartan as a combination for angiotensin receptor neprilysin inhibition (ARNI) have demonstrated a decrease in the mortality rate as compared to angiotensin receptor blocker alone [1]. Moreover, the Prospective Comparison of Sacubitril/Valsartan with ACEI to Determine Impact on Global Mortality and Morbidity in Heart Failure (PARADIGM-HF) study showed a significant improvement of left ventricular ejection fraction (LVEF) in patients suffering from heart failure with reduced ejection fraction (HFrEF) as compared to angiotensin converting enzyme inhibitors [2]. 

In the PARADIGM-HF trial, sudden cardiac death (SCD) was more decreased in the sacubitril/valsartan as compared with ACEI. A further prospective study recruited patients with HFrEF, treated with sacubitril/valsartan, and compared them with the treatment data on ACEI and/or angiotensin receptor blocker (ARB) [3]. The occurrence of nonsustained ventricular tachycardia (nsVT) and the premature ventricular contraction (PVC) burden were less frequent with sacubitril/valsartanin treatment in this single-center study [3]. However, recently published data presented six cases of ventricular arrhythmic storm shortly after initiating sacubitril/valsartan that required drug withdrawal [4]. However, no systematic analysis of the incidence of ventricular tachyarrhythmias in patients treated with sacubitril/valsartan in a sufficient number of patients with long-term follow-up has been conducted yet.

Given the limited evidence of the impact of sacubitril/valsartan treatment on the incidence of ventricular tachyarrhythmias in patients with chronic heart failure, we conducted a retrospective analysis in patients treated with sacubitril/valsartan at the University Medical Center Mannheim with systematic, long-term follow-up.

## 2. Methods

**Figure 1:**
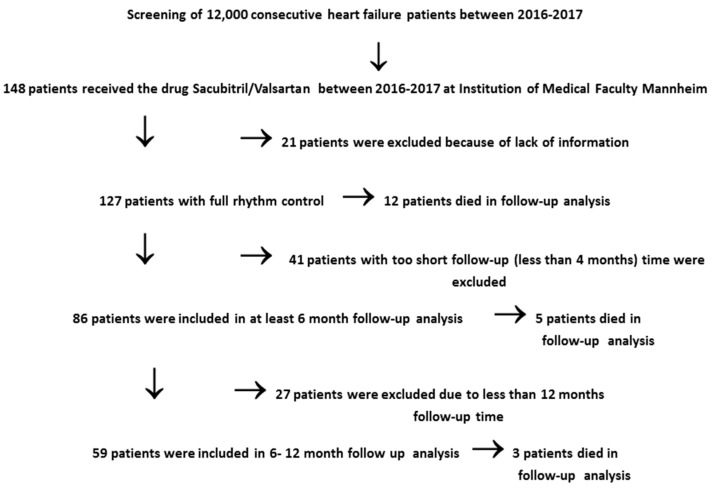
Flowchart presenting the screened data and included patients for the present study.

Out of 12,000 patients with HFrEF from 2016–2018, 148 consecutive patients were initiated on sacubitril/valsartan, and whole data of 127 patients were available (Figure 1). Patients were diagnosed according to the European Society of Cardiology (ESC) Guidelines for heart failure [5]. Inclusion criteria were (1) heart failure symptoms with New York Heart Association (NYHA) functional class ≥II despite optimal medical treatment (including angiotensin-converting enzyme inhibitor or angiotensin-II receptor blocker, beta blockers, and mineral-corticoid antagonist if tolerated); (2) LVEF ≤ 40%; (3) implanted ICD (implantable cardioverter defibrillator), CRT (cardiac resynchronization therapy), pacemaker, and/or loop recorder; and (4) patient received and tolerated sacubitril/valsartan.

A total of 59 out of 127 patients were completely followed for 12 months regarding ventricular arrhythmias (ventricular tachycardia (VT), non-sustained ventricular tachycardia (nsVT), ventricular fibrillation (VF)). The clinical outcome of patients was assessed by chart review and/or telephone review. At follow-up, cardiac device interpretation was carried out by two experienced electrophysiologists.

In patients with sacubitril/valsartan intolerance or relevant side effects (allergic reaction with angioedema, relevant decrease in blood pressure, depressed kidney function, and hyperkalemia), treatment was terminated. Different laboratory parameters were evaluated, including NT-brain natriuretic protein (NT-BNP), creatine kinase (CK), creatine kinase of myocardial type (CKMB), kidney parameters (creatinine and glomerular filtration rate (GFR)), before and after initiation of sacubitril/valsartan. Additionally, electrogcardiography (ECG) intervals, including the QTc interval, heart rhythm, and blood pressure, were documented. To qualify the effects of sacubitril/valsartan, echocardiography was frequently outlined in addition to clinical evaluation before and after sacubitril/valsartan during subsequent clinical visits. 

This study was conducted in compliance with the Declaration of Helsinki regarding investigations in human subjects, and the study protocol was approved by the Ethics Committee of the Medical Faculty Mannheim (2018-851R-MA). 

## 3. Statistics

Data are presented as means ± standard deviation for continuous variables with a normal distribution, or median (interquartile range) for continuous variables with a non-normal distribution or frequency (%) for categorical variables. The Kolmogorov–Smirnov test was used to assess normal distribution. Student’s *t* test and the Mann–Whitney U-test were used to compare continuous variables with normal and non-normal distributions, respectively. The chi-squared test or Fisher’s exact test was used to compare categorical variables. For paired non-parametric quantitative variables, a Wilcoxon signed-rank test was used. A McNemar test was used for paired qualitative variables. The cumulative probability of survival free from ventricular tachyarrhythmias was determined by Kaplan–Meier analysis. Statistical analysis was performed using SPSS 23.0 (Armonk, NY, USA: IBM Corp). 

## 4. Results

### 4.1. Characteristics of Patients Started on Sacubitril/Valsartan Treatment

Baseline characteristics are outlined in Table 1 and Table 2. Ischemic heart failure was diagnosed in 53% of patients. Patients with an age of 67 ± 12.11 years had a median LVEF of 25% (IQR 5.00–45.00) before sacubitril/valsartan and a median LVEF of 30% (IQR 10.00–55.00, *p* < 0.0005) after sacubitril/valsartan treatment, respectively. Systolic and diastolic blood pressure decreased from 127.93 ± 22.01 mmHg to 118.36 ± 20.55 mmHg (*p* = 0.0035). The echocardiography of aortic and mitral valve regurgitation was not significantly influenced by sacubitril/valsartan. However, tricuspid regurgitation tended to be reduced (moderate valve regurgitation from 17.9% to 11.9% after sacubitril/valsartan and severe regurgitation from 13.43% to 11.94% in patients, *p* = 0.07). The concomitant application of drugs, which were well balanced before and after treatment with sacubitril/valsartan, is presented in Table 1 and Table 2. 

### 4.2. Incidence of Ventricular Arrhythmia after Sacubitril/Valsartan Use

**Figure 2:**
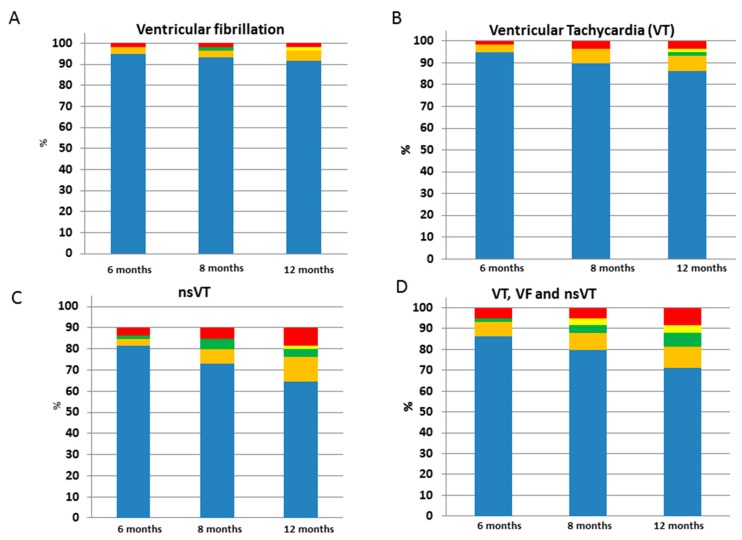
Comparison of ventricular arrhythmias before and after initiation of sacubitril/valsartan (**A**) Ventricular fibrillation (VF), (**B**) Ventricular tachycardia (VT), (**C**) Non-Sustained VT (nsVT), (**D**) A composite of VT, VF and nsVT. Red, more than 3 events; Yellow, 3 events; Green, 2 events; Orange, 1 event; Blue, no events.

**Figure 3:**
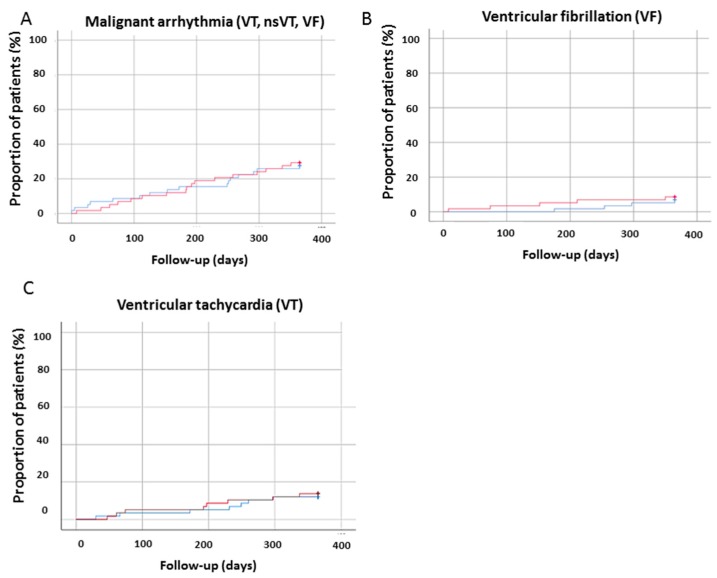
The incidence of ventricular arrhythmias of patients over 12 months of follow-up (before and after sacubitril/valsartan). The red line represents the data after sacubitril/valsartan treatment, the blue line represents the data before sacubitril/valsartan treatment. (**A**) A composite of ventricular tachycardia (VT), non-sustainded (nsVT) and ventricular fibrillation (VF), **(B**) VF, (**C**) VT.

In 59 patients, long-term outcomes at 6, 8, and 12 months were evaluated (Table 3, Table 4 and Table S1). Although the incidence of ventricular arrhythmia increased over 12 months of follow-up (at 6 months 15% versus 8.4%, at 8 months 15% versus 20.3%, and at 12 months 15% versus 28.8%) after initiation sacubitril/valsartan, the incidence of ventricular arrhythmias presented similar results when the data were compared 12 months before and 12 months after the initiation of sacubitril/valsartan (Figure 2 and Figure 3). After a follow-up of 12 months, only three patients died (5%).

To understand these findings (increased incidence of ventricular arrhythmias after 12 months of follow-up compared with initiation of sacubitril/valsartan), we separated patients according to ischemic and nonischemic cardiomyopathy. Whereas in patients with nonischemic cardiomyopathy ventricular arrhythmia events were similar before and after sacubitril/valsartan, these events were higher in ischemic cardiomyopathy patients (11% versus 37%, *p* = 0.04; Table S2).

## 5. Discussion

Our findings of the present retrospective analysis on 127 patients treated with sacubitril/valsartan are: (1) sacubitril/valsartan improved the LVEF in HFrEF patients and (2) sacubitril/valsartan failed to reduce the risk of ventricular arrhythmias after 12 months of follow-up. 

Sacubitril/valsartan is recommended (class I) as a replacement for an ACEI in patients with chronic heart failure and symptoms, although optimal medical treatment of heart failure is needed to further reduce the risk of hospitalization and death [6,7]. 

To date, sacubitril/valsartan has reduced the risk of SCD from 1.8 to 1.5% in the PARADIGM study and ventricular tachycardia from 2 to 1.5% as well [2]. However, these data could not be explained by the study group. 

Of note, the MADIT trial showed a significant reduction of SCD in patients who were suffering from ischemic cardiomyopathy and carrying an ICD [8]. Patients with a prior myocardial infarction and LVEF < 35%, and with an asymptomatic, nonsustained ventricular tachycardia, showed a significant reduction in all-cause mortality and cardiovascular mortality during 27 months of follow-up by using an ICD. Despite all advantages during last three decades in the diagnosis and therapy of patients with HFrEF, the outcomes remain poor and are dominated by SCD. 

Recently published data showed a significant reduction of ventricular arrhythmias in HFrEF patients treated with sacubitril/valsartan [3]. Furthermore, appropriate ICD shocks were significantly decreased. That study followed patients for nine months, and the LVEF was increased over follow-up, and ventricular arrhythmia events were reduced. The underlying mechanism for these findings was speculated based on experimental studies. In vitro studies have shown that sacubitril/valsartan may suppress cardiac fibrosis and remodeling compared to enalapril [1,9]. Although, another possible related explanation for the reduction of arrhythmia events in patients with heart failure is an increase in potassium level, as the study group presented a significant increase in potassium level with increased arrhythmia events [10].

However, our study on patients treated with sacubitril/valsartan with a follow-up of 12 months presented a persistently increased incidence of ventricular arrhythmias and increased anti-tachycardia pacing and/or ICD shocks, although a significant improvement in the LVEF after the initiation of sacubitril/valsartan treatment was documented, which is consistent with published data. Additionally, in examinations using echocardiography, tricuspid regurgitation tended to be reduced after treatment with sacubitril/valsartan. The increased ventricular arrhythmia events might be explained by more than one hypothesis. Since the LVEF increased and patients were doing well, patients might tend to be noncompliant or infrequently intake their heart failure drugs. Although there was no significant difference between patients treated with aldosterone antagonists before and after initiation of sacubitril/valsartan, the number of patients was numerically lower after initiating the treatment with sacubitril/valsartan. Of note, an in-depth analysis of patients with a composite of VT, nsVT, and VF after sacubitril/valsartan treatment found that five patients did not receive aldosterone antagonists after initiation of sacubitril/valsartan. Further, five patients presented a long QTc interval, with a QTc > 500 ms in at least three of them. 

A further speculated hypothesis is the reduced scar size after initiation of sacubitril/valsartan. It might be that scar islands may be pro-arrhythmic with a higher susceptibility of ventricular arrhythmias. These pro-arrhythmic scars could be suppressed by anti-arrhythmic drugs concomitant with sacubitril/valsartan. Furthermore, although an increase in potassium levels after treatment with sacubitril/valsartan was documented, which is frequently related to lower arrhythmia events, it was not excluded that other electrolytes, such as low magnesium levels, might be related to the increased arrhythmia events. 

Therefore, after initiation of sacubitril/valsartan, frequent follow-ups with patients and proper control of their heart failure therapy might be important. Furthermore, the concomitant use of anti-arrhythmic drugs might be important to reduce the increased risk of ventricular arrhythmias in the sacubitril/valsartan group. Despite this careful evaluation, it seems that the risk of ventricular arrhythmias in patients with nonischemic cardiomyopathy is not increased as compared to that in patients with ischemic cardiomyopathy. It is well known that patients with ischemic heart failure suffer more frequently from malignant arrhythmias as compared to patients with nonischemic heart failure [10]. Therefore, these data support the indication of ICD implantation in nonischemic cardiomyopathy evaluated by the DANISH trial [11].

A further important finding is the observed impairment in renal function. Therefore, frequent evaluation of kidney function is essential in patients treated with sacubitril/valsartan. The impairment of renal function might be explained by a reduced blood pressure, which was observed in our patients. One important inclusion criterion of the PARADIGM trial was having a systolic blood pressure higher than 95 mmHg at randomization to overcome the hypotension effect of sacubitril/valsartan. Recently, published data have shown that, despite an increase in the albumin/creatinine ratio in the sacubitril/valsartan group as compared to that of the enalapril group, combination therapy displayed a slower decrease in the GFR and improved cardiovascular outcomes [12]. 

## 6. Conclusions

Despite promising data of sacubitril/valsartan use in chronic heart failure patients, combination therapy might not reduce ventricular arrhythmia events. Therefore, the concomitant use of anti-arrhythmic drugs might be important to reduce the risk of ventricular arrhythmias in sacubitril/valsartan treatment. Furthermore, care should be taken when terminating other heart failure therapies, although the LVEF has been improved. 

### 6.1. Study Limitations

This is a single-center study with a limited follow-up time. Patients were retrospectively evaluated. Despite novel findings of an increased LVEF and decreased tricuspid regurgitation, these data were not systematically evaluated using cardiac magnetic resonance. The NYHA class was evaluated subjectively without a qualitative evaluation using a questionnaire. At this moment, the reason for these findings cannot be explained. However, these data can be evaluated in multicenter, prospective studies. Whether arrhythmia events increase only in ischemic heart failure patients needs to be confirmed by further studies. 

### 6.2. Clinical Perspectives

Sacubitril/valsartan is a good treatment approach in HFrEF patients. However, caution is required because sacubitril/valsartan might increase the risk of ventricular arrhythmias over time despite the improvement in the left ventricular ejection fraction.

## Figures and Tables

**Table 1:** Baseline characteristics of the 127 patients initially presenting before and after sacubitril/valsartan.

Variables	Before Sacubitril/Valsartan *n* = 127	After Sacubitril/Valsartan *n* = 127	*p* Value *
**Demographics**			
BMI; median – IQR (min–max)	30 (17.5–49.5)	30 (17.5–64.20)	0.04
**Clinical parameters**			
Systolic BP; mmHg; mean ± SD	127.93 ± 22.01	118.36 ± 20.55	0.0035
Diastolic BP; mmHg, median – IQR (min–max)	80 (42–110)	70 (30–113)	0.01
Heart rate; bpm, median – IQR (min–max)	76 (51–140)	70 (48–131)	0.009
**Laboratory values**			
GFR (mL/min); median – IQR (min–max)	55.50 (21.00–137.00)	47.00 (13.00–116.00)	0.83
Creatinine (mg/dl); (median – IQR (min–max)	1.31 (0.64–3.55)	1.45 (0.10–7.10)	0.32
Alanine transaminase (U/L); median – IQR (min–max)	25 (5.90–707.00)	24 (7.00–2532.00)	0.34
Aspartate transaminase (U/L); median – IQR (min–max)	23 (11.00–1147.00)	21 (10.00–6866.00)	0.03
Gamma-glutamyltransferase (U/L); median – IQR (min–max)	50.50 (14–488)	45 (10–913)	0.06
Bilirubin (mg/dl); median – IQR (min–max)	0.59 (0.07–2.74)	0.52 (0.21–5.22)	0.28
Potassium (mmol/l); mean ± SD	4.17 ± 0.57	4.37 ± 0.65	0.0014
LDH; median – IQR (min–max)	227 (88–1776)	223 (156–5550)	0.93
TNI (µg/L); median – IQR (min–max)	0.08 (0.01–103)	0.01 (0.01–44.00)	0.15
CK (U/L); median – IQR (min–max)	89 (31–971)	74 (28–624)	0.18
CK-MB (U/L); mean ± SD	19 (5–55)	16 (13–27)	1.00
proBNP (ng/L); median – IQR (min–max)	1778 (100–74,676)	1248 (12.9–25,877)	0.27
Hemoglobin (g/dl); median – IQR (min–max)	13.5 (8.2–16.8)	13.7 (7.7–18.9)	0.94
C-reactive protein (mg/L); median – IQR (min–max)	5.00 (0.90–100)	6.4 (0.2–183)	0.75
HBA1c (%); median – IQR (min–max)	7.00 (4.9–12)	6 (5–8.10)	0.21
**ECG Data. Mean ± SD**			
PQ time	176.85 ± 33.79	179.43 ± 41.88	0.78
QT time	433.44 ± 60.73	442.48 ± 45.67	0.40
QTc time	475.38 ± 48.48	471.36 ± 39.47	0.33
**Medical history. *n* (%)**			
Smoking	22 (23.9)	18 (19.6)	0.16
Diabetes mellitus type 2	43 (35.8)	45 (37.50)	0.69
Hypertension	77(71.96)	79 (73.8)	0.59
COPD	17 (14.6)	19 (16.3)	0.41
Asthma	1 (0.9)	1 (0.9)	1.00
History of malignancy	11 (9.67)	11 (9.67)	1.00
Stroke	10 (8.7)	11 (9.56)	0.56
Bleeding	4 (3.36)	3 (2.52)	0.65
Ischemic CMP	53 (53)	53 (52)	1.00
**NYHA-Classification**			
1	0 (0.00)	7 (10.14)	0.22
2	20 (28.99)	20 (28.99)	0.22
3	43 (62.31)	36 (52.17)	0.22
4	6 (8.7)	6 (8.7)	0.22
**Echocardiography data. *n* (%)**			
EF(%); median – IQR (min–max)	25 (5.00–45.00)	30 (10.00–55.00)	<0.0005
Mitral valve regurgitation			
0 = none	12 (14.46)	13 (15.66)	0.26
1 = mild	27 (32.53)	34 (40.96)	0.26
2 = moderate	23 (27.71)	20 (24.1)	0.26
3 = severe	21 (25.3)	16 (19.28)	0.26
**Tricuspid valve regurgitation**			
0 = none	25 (37.31)	22 (32.83)	0.07
1 = mild	21 (31.34)	29 (43.28)	0.07
2 = moderate	12 (17.91)	8 (11.94)	0.07
3 = severe	9 (13.43)	8 11.94)	0.07
**Aortic valve regurgitation**			
0 = none	51 (79.68)	51 (79.69)	0.25
1 = mild	7 (10.93)	11 (10.94)	0.25
2 = moderate	6 (9.38)	2 (3.13)	0.25
**Electronic cardiac device**			
CRT	36 (29.03)	44 (35.48)	0.0047
ICD	71(57.72)	79 (64.22)	0.02
SM	7 (5.6)	8 (6.4)	0.56
CCM	28 (22.58)	35 (28.23)	0.05
Vagus stimulation	1 (0.826)	1 (0.826)	1.00

* *p* values for the comparison before and after sacubitril/valsartan; SD. Standard deviation; ECG. Electrocardiogram; EF. Ejection fraction; BMI. Body mass index. GFR. Glomerular filtration rate; ACE. Angiotensin-converting enzyme; CRT. Cardiac resynchronization therapy; CCM. Cardiac contractility modulation.

**Table 2:** Treatment of 127 patients before and after initiation of sacubitril/valsartan.

Variables	Before Sacubitril/Valsartan *n* = 127	After Sacubitril/Valsartan *n* = 127	*p* Value *
**Drugs on admission. *n* (%)**			
**Antidiabetic drugs**			
Metfomin	11 (8.94)	11 (8.94)	1.00
Insulin	19 (15.32)	17 (13.71)	0.32
DPP-4 Inhibitor	11 (8.94)	10 (8.13)	0.65
Sulfonylureas	4 (12.9)	0 (0.00)	0.05
SGLT2 Inhibitor	1 (0.81)	5 (4.07)	0.10
GLP-1 Agonist	0 (0.00)	1 (0.8)	0.32
Beta Blocker	114 (95.7)	116 (97.5)	0.48
AT-II Antagonist	32 (26.7)	0 (0.00)	<0.0001
Aldosterone Antagonist	86 (71.1)	88 (72.7)	0.71
Spironolactone	55 (46.4)	25 (21.07)	0.35
Eplerenone	30 (25.4)	39 (33.1)	0.03
ACE Inhibitor	67 (58.8)	2 (1.75)	<0.0001
Ramipril	67 (52.7)	0 (0.00)	<0.0001
Enalapril	3 (2.7)	0 (0.00)	0.08
Lisinopril	2 (2.7)	0 (0.00)	0.16
Diuretics	101 (82.8)	105 (86.1)	0.16
Hydrochlorothiazide	16 (13.3)	19 (15.8)	0.47
Xipamid	2 (1.67)	2 (1.67)	1.00
Torasemid	91 (75.2)	95 (78.5)	0.25
Furosemid	6 (4.96)	6 (4.96)	1.00
Platelet aggregation inhibitors	57 (47.5)	53 (44.2)	0.43
Ticagrelor	4 (3.31)	1 (0.83)	0.08
Aspirin	51 (42.1)	47 (38.8)	0.39
Clopidogrel	17 (14.4)	16 (13.6)	0.78
Prasugrel	3 (2.48)	1 (0.83)	0.16
Anticoagulation	58 (47.9)	63 (52.1)	0.20
Warfarin	27 (25.96)	26 (25)	0.74
Dabigatran	8 (6.72)	10 (8.4)	0.16
Edoxaban	1 (0.85)	1 (0.85)	1.00
Rivaroxaban	11 (9.24)	9 (7.56)	0.41
Apixaban	10 (8.47)	17 (14.4)	0.02
Statin	79 (66.9)	83 (70.33)	0.32
**Anti-arrhythmic drugs**			
Amiodarone	19 (15.7)	26 (21.5)	0.09
Sotalol	0/121 (0)	0 (0)	1.00
Mexiletine	0 (0)	1 (0.83)	1.00

* *p* values for the comparison before and after sacubitril/valsartan; SD. Standard deviation; ECG. Electrocardiography; EF. Ejection fraction; BMI. Body mass index. GFR. Glomerular filtration rate; ACE. Angiotensin-converting enzyme; CRT. Cardiac resynchronization therapy; CCM. Cardiac contractility modulation.

**Table 3:** Baseline characteristics of 59 patients initially presenting before and after sacubitril/valsartan.

Variables	Before Sacubitril/Valsartan *n* = 59	After Sacubitril/Valsartan *n* = 59	*p* Value *
BMI > 25 kg/m². mean ± SD	30 (17.8–40.8)	30.4 (17.47–49)	0.01
**Clinical parameters**			
Systolic BP; mmHg; mean ± SD	129 ± 21	121 ± 18	0.05
Diastolic BP; mmHg; median – IQR (min–max)	78 (53–100)	70 (50–113)	0.34
Heart rate. Bpm; median – IQR (min–max)	74 (55–115)	69 (48–131)	0.95
**Laboratory values**			
GFR (ml/min); median – IQR (min–max)	57.5 (25.0–105.0)	47.8 (15.0–100)	0.88
Creatinine (mg/dl); median – IQR (min–max)	1.38 (0.80–3.55)	1.4 (0.10–7.1)	0.41
Alanine transaminase (U/L); median – IQR (min–max)	24 (13–193)	24 (10–52)	0.74
Aspartate transaminase (U/L); median – IQR (min–max)	24 (12–80)	22 (10–73)	0.48
Gamma-GT (U/L); median – IQR (min–max)	58 (15–280)	55 (15–261)	0.09
Bilirubin (mg/dl); median – IQR (min–max)	0.45 (0.07–1.59)	0.48 (0.25–1.25)	0.65
Potassium (mmol/l); mean±SD	4.14 (3.00–5.40)	4.42 (3.10–5.50)	0.08
LDH; median – IQR (min–max)	226 (88–353)	241 (165–305)	0.51
TNI (µg/L); median – IQR (min–max)	0.093 (0.01–103)	44 (44–44)	0.32
CK (U/L); median – IQR (min–max)	96 (31–249)	81 (28–432)	0.87
proBNP (ng/L); median – IQR (min–max)	1735 (156–74676)	1845 (43–25877)	0.18
Hemoglobin (g/dl); median – IQR (min–max)	13.3 (8.2–16.8)	13.95 (8.3–18.9)	0.55
C-reactive protein (mg/L); median – IQR (min–max)	7.8 (2.4–100)	6.5 (0.40–181)	0.97
HBA1c (%); median – IQR (min–max)	7 (5–8)	6.1 (5.00–8.1)	0.23
**ECG Data.**			
PQ-Time; median – IQR (min–max)	184 (142–230)	178 (136–278)	1.00
QT-Time; median – IQR (min–max)	422 (334–508)	436 (354–548)	0.48
QTc-time; median – IQR (min–max)	465 (396–555)	455 (416- 552)	0.04
**Medical history. *n* (%)**			
Smoking	11/47 (23.4)	9/47 (19.1)	0.41
Diabetes mellitus type 2	18/56 (32.14)	21/56 (37.50)	0.25
Hypertension	40/51 (78.4)	42/51 (82.3)	0.41
COPD	9/53 (16.98)	11/53 (20.8)	0.32
Asthma	0/51 (0)	0 /51 (0)	1.00
History of malignancy	4/53 (7.55)	5/53 (9.43)	0.56
Stroke	5/55 (9.09)	5/55 (9.09)	1.00
Bypass	10/56 (17.85)	10/56 (17.85)	1.00
Bleeding	4/56 (7.14)	2/56 (7.14)	0.32
Ischemic cardiomyopathy	29/48 (60.41)	29/48 (60.41)	0.32
**NYHA-Classification**			
1	0/34 (0)	3/34 (8.82)	0.81
2	12/34 (35.29)	11/34 (32.35)	0.81
3	19/34 (55.89)	17/34 (0.50)	0.81
4	3/34 (8.82)	3/34 (8.82)	0.81
**Echocardiography data. *n* (%)**			
EF(%)	26 (15–40)	30 (14–55)	0.10
**Mitral valve regurgitation**			
0 = none	5/35 (14.28)	5/35 (14.28)	0.76
1 = mild	11/35 (31.42)	12/35 (34.29)	0.76
2 = moderate	9/35 (25.71)	9/35 (25.71)	0.76
3 = severe	10/35 (28.57)	9/35 (25.71)	0.76
**Tricuspid valve regurgitation**			
0 = none	11/27 (40.74)	10/27 (37.04)	0.91
1 = mild	10/27 (37.04)	12/27 (44.44)	0.91
2 = moderate	2/27 (7.04)	2/27 (7.04)	0.91
3 = severe	4/27 (14.81)	3/27 (11.11)	0.91
**Aortic valve regurgitation**			
0 = none	23/26 (88.46)	21/26 (80.77)	0.57
1 = mild	1/26 (3.85)	4/26 (15.38)	0.57
2 = moderate	2/26 (7.69)	1/26 (3.85)	0.57
**Cardiac electronic device**			
CRT	11/57 (19.30)	14/57 (24.56)	0.08
ICD	38/57 (66.67)	41/57 (71.93)	0.18
SM	2/58 (3.45)	1/58 (1.72)	0.32
CCM	21/57 (36.84)	24/57 (42.11)	0.32
Vagus stimulator	0/58 (0)	0/58 (0)	1.00

* *p* values for the comparison before and after sacubitril/valsartan; SD. Standard deviation; ECG. Electrocardiogram; EF. Ejection fraction; BMI. Body mass index. GFR. Glomerular filtration rate; ACE. Angiotensin-converting enzyme; CRT. Cardiac resynchronization therapy; CCM. Cardiac contractility modulation.

**Table 4:** Treatment of 59 patients before and after initiation of sacubitril/valsartan.

Variables	Before Sacubitril/Valsartan *n* = 59	After Sacubitril/Valsartan *n* = 59	*p* Value *
**Drugs on admission. *n* (%)**			
Antidiabetic therapy			
Metfomin	6/58 (10.34)	7/58 (12.07)	0.65
Insulin	10/58 (17.24)	7/58 ((12.07)	0.08
DPP-4 Inhibitor	4/58 (6.90)	5/58 (8.62)	0.56
Sulfonylureas	3/58 (5.17)	0/58 (0)	0.08
SGLT2 Inhibitor	0/58 (0)	5/58 (8.62)	0.03
-GLP-1-Agonist	0/58 (0)	1/58 (1.72)	0.32
Beta Blocker	56/57 (89.24)	56/57 (98.25)	1.00
AT-II Antagonist	17/58 (29.31)	0/58 (0)	<0.001
Aldosterone Antagonist	46/58 (79.31)	44/58 (75.86)	0.59
Aldactone	29/57 (50.80)	20/57 (35.08)	0.38
Eplerenone	17/57 (29.82)	24/57 (42.11)	0.035
ACE Inhibitor	30/57 (52.63)	0/57 (0)	<0.001
Ramipril	31/55 (56.3)	0/55 (0)	<0.001
Enalapril	1/56 ((1.78)	0/56 (0)	0.32
Lisinopril	0/55 (0)	0/55 (0)	1.00
Diuretics	50/58 (86.20)	53/58 (91.38)	0.08
Hydrochlorothiazide	8/57 (14.04)	8/58 (13.79)	1.00
Xipamid	0/57 (0)	1/57 (1.75)	0.32
Torasemid	46/58 (79.31)	49/58 (84.48)	0.26
Furosemid	2/58 (3.45)	3/58 (5.17)	0.56
Platelet aggregation inhibitors	35/58 (0.60)	32/58 (55.17)	0.44
Brilique	1/58 (1.72)	0/58 (0)	0.32
Aspirin	31/58 (53.45)	29/58 (50)	0.59
Clopidogrel	10/56 (17.86)	8/56 (14.29)	0.41
Prasugrel	3/59 (5.08)	1/59 (1.69)	0.16
Anticoagulation	21/58 (36.21)	25/58 (43.10)	0.16
Warfarin	11/48 (22.92)	12/48 (25)	0.56
Dabigatran	3/56 (5.36)	5/56 (8.93)	0.16
Edoxaban	0/56 (0)	0/56 (0)	1.00
Rivaroxaban	3/56 (5.36)	3/56 (5.36)	1.00
Apixaban	4/57 (7.02)	8/57 (14.04)	0.10
Statin	40/56 (41.43)	43/56 (76.79)	0.41
**Antiarrhythmic drugs**			
Amiodarone	9/57 (15.79)	15/57 (26.31)	0.07
Sotalol	0/57 (0)	0/57 (0)	1.00
Mexiletine	0/57 (0)	1/57 (1.75)	1.00

* *p* values for the comparison before and after sacubitril/valsartan; SD. Standard deviation; ECG. Electrocardiogram; EF. Ejection fraction; BMI. Body mass index. GFR. Glomerular filtration rate; ACE. Angiotensin-converting enzyme; CRT. Cardiac resynchronization therapy; CCM. Cardiac contractility modulation.

## Supplementary Materials

The following are available online at https://www.mdpi.com/2077-0383/8/10/1582/s1, Table S1: Ventricular arrhythmia events of patients over 12 months before and after treatment with sacubitril/valsartan, Table S2: Comparison of life-threatening arrhythmia events in the presence and absence of ischemic cardiomyopathy.
