# Supplementary material for: Impact of Sacubitril/Valsartan on the Long-Term Incidence of Ventricular Arrhythmias in Chronic Heart Failure Patients
Source: J Clin Med. 2019 Oct 2;8(10):1582. doi: 10.3390/jcm8101582 (PMC6832713; doi:10.3390/jcm8101582)
Supplement: Supplementary file 1 [file jcm-08-01582-s001.pdf]

Table S1. Ventricular arrhythmia events of patients treated with sacubitril/valsartan over 12 months.

Variables	Before Sacubitril/Valsartan *n* = 59	After Sacubitril/Valsartan *n* = 59	*p* Value *
**Arrhythmias, n (%)**			
Composite of VT, nsVT and VF	9 (15)	18 (30.5)	0.01
After 6 months	9 (15)	8 (8.42)	0.78
After 8 months	9 (15)	12 (20.3)	0.32
After 12 months	9 (15)	17 (28.8)	0.03
**Ventricular fibrillation**			
After 6 months	1 (1.7)	3 (5.08)	0.32
After 8 months	1 (1.7)	4 (6.78)	0.18
After 12 months	1 (1.7)	5 (8.47)	0.10
**nsVT**			
After 6 months	4 (6.78)	5 (8.47)	0.71
After 8 months	4 (6.78)	10 (16.9)	0.03
After 12 months	4 (6.78)	14 (23.7)	0.0039
**Ventricular tachycardia**			
After 6 months	4 (6.78)	3 (5.08)	0.65
After 8 months	4 (6.78)	6 (10.2)	0.48
After 12 months	4 (6.78)	8 (8.42)	0.21
**Rhythm therapy (ATP; Shock); n (%)**			
After 6 months	4 (6.78)	5 (8.47)	1.00
After 8 months	4 (6.78)	8 (13.6)	0.34
After 12 months	4 (6.78)	10 (16.9)	0.15

* *p* values for the comparison before and after sacubitril/valsartan, VT = ventricular tachycardia, nsVT = non-sustained ventricular tachycardia, VF = ventricular fibrillation, ATP = antitachycardia pacing.

Table S2. Comparison of life-threatening arrhythmia events in presence or absence of ischemic cardiomyopathy.

Variables	Before Sacubitril/Valsartan *n* = 59	After Sacubitril/Valsartan *n* = 59	*p* Value *
**Ischemic cardiomyopathy; *n* = 30**			
**Malignant Arrhythmia (VF, nsVT, VT) *n* (%)**			
After 6 months	5/30 (16.6)	6/30 (20.0)	1.00
After 8 months	5/30(16.6)	7/30 (23.3)	0.73
After 12 months	5/30(16.6)	12/30 (40.0)	0.09
**Ventricular fibrillation** *n* (%)			
After 6 months	0/30 (0)	3/30 (10.0)	0.25
After 8 months	0/30 (0)	3/30 (10.0)	0.25
After 12 months	0/30 (0)	4/30 (13.3)	0.13
**nsVT** *n* (%)			
After 6 months	3/27 (11.1)	4/27 (14.8)	1.00
After 8 months	3/27 (11.1)	6/27 (22.2)	0.38

After 12 months	3/27 (11.1)	10/27 (37.0)	0.04
**Ventricular tachycardia** *n* (%)			
After 6 months	2/30 (6.67)	2/30 (6.67)	1.00
After 8 months	2/30 (6.67)	3/30 (10.0)	1.00
After 12 months	2/30 (6.67)	5/30 (16.7)	0.45
**Non-Ischemic cardiomyopathy; *n* = 29**			
**Malignant arrhythmia (VF, nsVT, VT) *n* (%)**			
After 6 months	4/29 (13.8)	2/29 (6.90)	0.63
After 8 months	4/29 (13.8)	5/29 (17.2)	1.00
After 12 months	4/29 (13.8)	5/29 (17.2)	1.00
**Ventricular fibrillation** *n* (%)			
After 6 months	1/29 (3.45)	0/29 (0.00)	1.00
After 8 months	1/29 (3.45)	1/29 (3.45)	1.00
After 12 months	1/29 (3.45)	1/29 (3.45)	1.00
**nsVT** *n* (%)			
After 6 months	1/17 (5.88)	1/17 (5.88)	1.00
After 8 months	1/17 (5.88)	4/17 (23.5)	0.25
After 12 months	1/17 (5.88)	4/17 (23.5)	0.25
**Ventricular tachycardia** *n* (%)			
After 6 months	2/29 (6.90)	1/29 (3.45)	1.00
After 8 months	2/29 (6.90)	3/29 (10.3)	1.00
After 12 months	2/29 (6.90)	3/29 (10.3)	1.00

* *p* values for the comparison before and after sacubitril/valsartan. VT = ventricular tachycardia, nsVT = non-sustained ventricular tachycardia, VF = ventricular fibrillation.
